# Is *Amanita phalloides* Nephrotoxicity due to Mitochondrial Toxicity?

**DOI:** 10.1016/j.xkme.2024.100952

**Published:** 2024-12-24

**Authors:** Jules Weinhard, Justine Serre, Perrine Frère, Clovis Adam, Marie Camille Lafargue, David Buob, Cédric Rafat

**Affiliations:** 1 Service de Néphrologie, Dialyse, Aphérèses et Transplantation Rénale, CHU Grenoble Alpes, La Tronche, France; 2 Soins Intensifs Néphrologiques et Rein Aigu, Hôpital Tenon, AP-HP, Paris, France; 3 Inserm UMRS 1155, Department of Pathology, Sorbonne Université, Hôpital Tenon, AP-HP, 75020 Paris, France; 4 Anatomie et Cytologie Pathologiques, CHU Bicêtre, AP-HP, Paris, France; 5 Anatomie et cytologie pathologiques, Hôpital Tenon, AP-HP, Paris, France; 6 French Intensive Renal Network, Lyon, France

**Keywords:** Acute kidney injury, acute tubular injury, chronic kidney disease, cytochrome C oxidase–succinate dehydrogenase (COX-SDH), estimated glomerular filtration rate, organic anion transporting polypeptides 1B3 (OATP1B3), retinol-binding protein (RBP), translocase outer membrane 20 (TOM20)

## Abstract

*Amanita phalloides*-related kidney toxicity is poorly documented and remains to be elucidated. Herein, we describe the case of a 43-year old patient who presented with severe liver failure following the ingestion of *Amanita phalloides*. Although liver injury subsided following the administration of N-acetyl cystein and silibinin, the patient subsequently developed KDIGO stage 3 acute kidney injury. Histopathological examination of the kidney displayed moderate tubular injury characterized by dilated tubular lumens and flattening of the tubular epithelium on optic microscopy. Electron microscopy showed mitochondrial changes including swelling and decreased number of cristae. Immunofluorescence for the key mitochondrial protein TOM20 found significantly decreased expression compared with ischemic acute tubular injury. Despite these changes, histoenzymology showed preserved succinate cytochrome c oxidase (COX) expression, suggesting that mitochondrial complex IV function was maintained. Our findings suggest that *Amanita phalloides* elicits acute tubular injury via mitochondrial damage, possibly through a pathway that spares COX function.

*Amanita phalloides*, commonly known as the death cap mushroom and prevalent in Central, Western Europe and North America, poses a growing concern in our digital era. The increasing reliance on smartphone applications for mushroom identification has contributed to an increase in poisoning cases.^1^ Toxicity is mainly attributed to amatoxins (particularly α-amanitin), which are resistant to cooking, freezing, gastric acidity, and gastrointestinal enzymes.

Poisoning by *Amanita phalloides* exhibits a 3-stage clinical course, including the gastrointestinal phase (6-24 hours after ingestion), the latency phase (lasting 24-72 hours), and finally the hepato-renal phase sets in beyond 72 hours and is characterized by the full-blown clinical manifestations of acute liver failure and kidney injury (AKI).

Although liver toxicity is well documented, the mechanisms of nephrotoxicity are poorly understood, even though AKI occurs in 10% to 18% of cases.^2^

## Case Report

We report the case of a 43-year-old man with no prior medical history who consulted for abdominal pain and emesis. He disclosed the ingestion of *Amanita phalloides* 72 hours prior admission. The identity of the ingested mushroom was confirmed on photographic verification at the poison center. Clinical examination was unremarkable: the patient’s blood pressure was recorded at 142/95 mm Hg and he did not exhibit sign of peripheral hypoperfusion. The biologic assessment showed plasma creatinine levels of 76 μmol/L, estimated glomerular filtration rate of 106 mL/min/1.73 m^2^ (Chronic Kidney Disease Epidemiology Collaboration), plasma potassium levels of 4.1 mmol/L, plasma sodium levels of 142 mmol/L, aspartate aminotransférase of 3,610 UI/L, alanine transaminase of 7,221 UI/L, prothrombin time of 54%, gamma-glutamil transpeptidase of 36 UI/L, alkaline phosphatase of 57 UI/L, total bilirubin levels of 36 μmol/L, and normoglycemia of 5.9 mmol/L.

Acute liver failure prompted the administration of N-acetyl cystein and OATP1B3 inhibitor (silibinin, Legalon) in addition to cristalloid volume expansion. He did not require vasopressor support at any time point. The course was swiftly favorable with respect to liver function tests, as transaminase levels and prothrombin time normalized at days 9 and 5 after poisoning, respectively. Despite the rapid correction of hypovolemia, AKI occurred starting on day 5 following intoxication, reaching a plateau of 316 μmol/L by day 9, without any criterion for kidney replacement therapy.

On diagnostic work-up, low-level proteinuria (0.34 g/24 h) associated with microalbuminuria (220 mg/g creatinine), alpha 1-microglobulin/creatininuria levels of 3.56 mg/mmol (N < 2.26) with no increase in retinol-binding protein, and transient microscopic hematuria were found. Kidney ultrasound examination was unremarkable. Serum protein immunofixation was normal. Human immunodeficiency virus, hepatitis B virus, hepatitis C virus, leptospirosis, and hantavirus serologic testing were negative.

## Results

**Figure 1:**
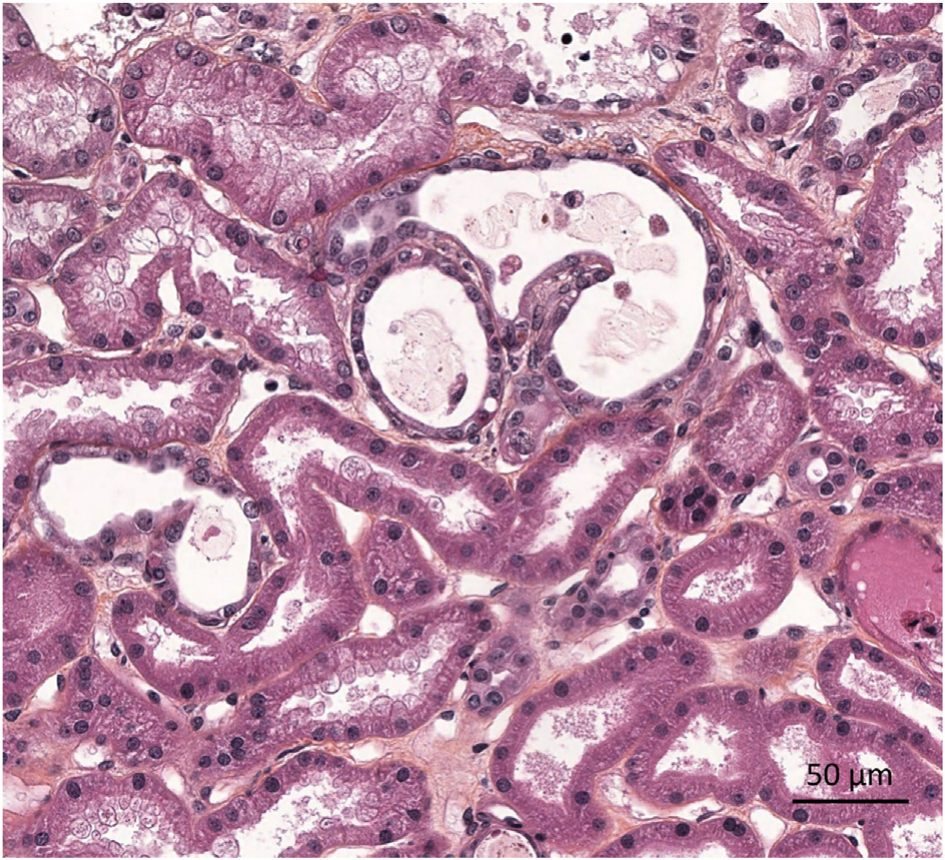
*Amanita phalloides*-related acute tubular necrosis and mitochondria damage. Optic microscopy displaying moderate tubular injury with dilated tubular lumens and flattening of the tubular epithelium. Scale bar: 50 μm.

**Figure 2:**
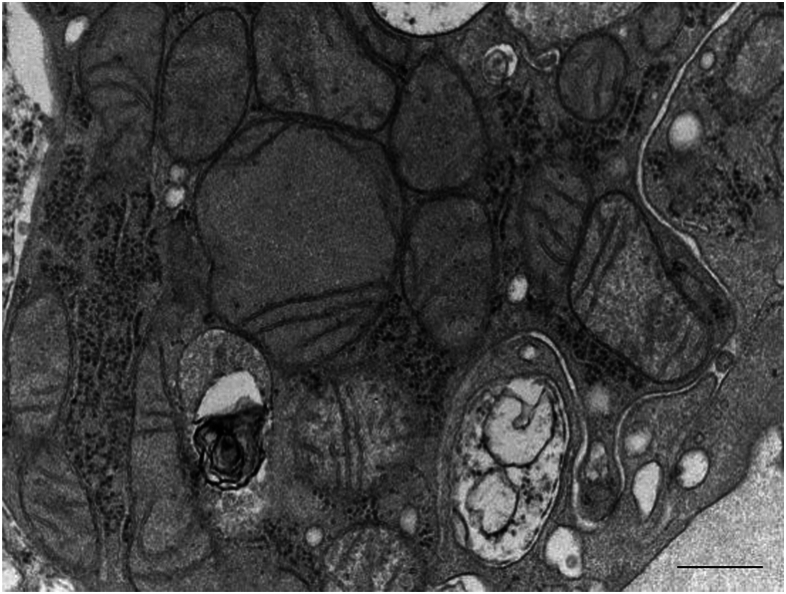
*Amanita phalloides*-related acute tubular necrosis and mitochondria damage. Electron microscopy showing mitochondrial changes including swelling and decreased number of cristae. Scale bar: 500 nm.

**Figure 3:**
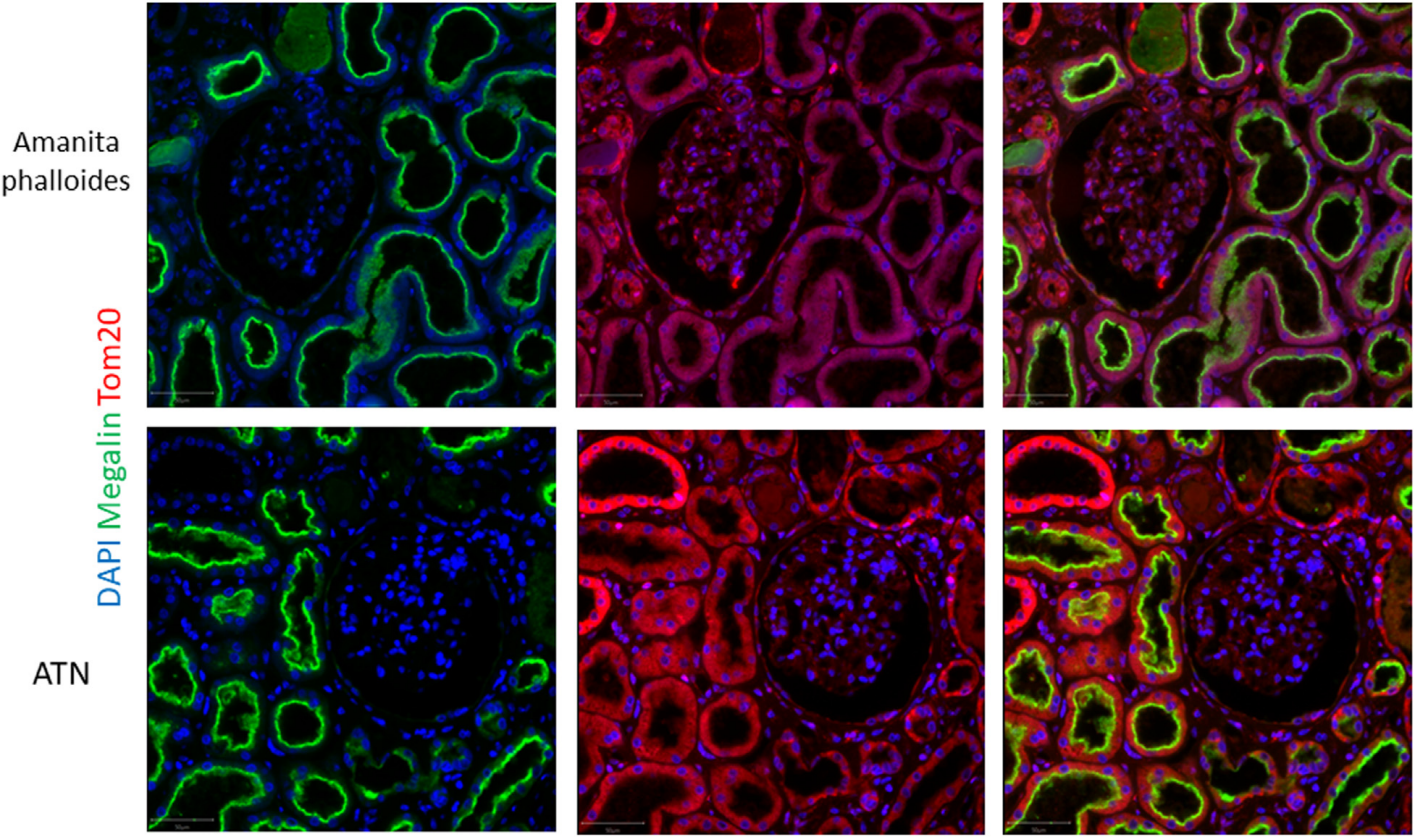
Functional histoenzymology on kidney sections for COX/SDH mitochondrial enzymes (double staining). Functional histochemistry on kidney sections for COX/SDH mitochondrial enzymes double staining. SDH activity, encoded by nuclear DNA, gives a blue color; COX activity, encoded by mitochondrial DNA and nuclear DNA, yields a brown color; and COX-SDH double staining exhibits a brown-blue color. Diffuse brown on all proximal tubule section, which displayed histological signs of acute tubular necrosis, indicating preserved COX activity reflecting preserved mitochondrial cytochrome c oxidase function.

**Figure 4:**
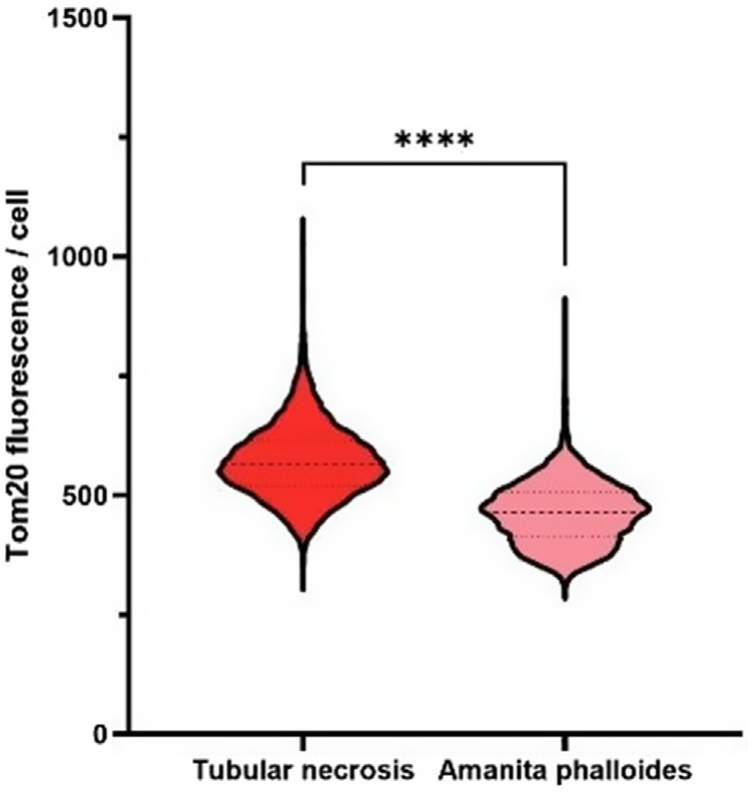
Decreased TOM20 kidney tubular expression after *Amanita phalloides* exposure. Immunostaining of TOM20 (red) and megalin (green) of kidneys from the patient with Amanita phalloides toxicity and a 33-year-old control patient with stage 3 KDIGO acute kidney injury with acute tubular necrosis because of septic and hemorrhagic shock. Scale bar: 50 μm.

The lack of kidney function recovery mandated a kidney biopsy on the 15th day postintoxication. Light microscopy analysis showed moderate tubular injury (Figure 1). Immunofluorescence showed no significant immune deposits. Electron microscopy was remarkable for mitochondrial changes including swelling and decreased number of cristae (Figure 2). Functional histoenzymology for mitochondrial respiratory chain enzymes cytochrome c oxidase (COX) and succinate dehydrogenase (SDH) activities found no evidence for reduced COX/SDH function in renal tubular cells, including in tubules displaying acute tubular injury (ATI) lesions (Item S1), indicating preserved cytochrome C oxidase activity. Kidney tubular expression of TOM20 (immunofluorescence) was significantly decreased (Figures 3 and 4).

No specific treatment was initiated, creatinine stabilized, allowing the patient be discharged home on the 17th day postintoxication.

The outpatient course was remarkable for gradual recovery with a follow-up creatinine of 114 μmol/L at month 4 (estimated glomerular filtration rate of 67 mL/min/1.73 m^2^).

## Discussion

In recent years, mortality rates attributed to *Amanita phalloides* poisoning have plummeted from 10% to 30% in historical studies to around 10% in the past 20 years, thanks to improved management combining (1) fluid expansion; (2) liver protection strategies that hinge both on N-acetyl-cysteine and the administration of silibinin, which inhibits OATP1B1 involved in α-amanitin entry into the liver; and (3) ultimately liver transplantation. Extrarenal purification techniques, such as hemodialysis or hemoperfusion, have not demonstrated significant clinical benefits, potentially because these treatments are initiated relatively late compared with the plasma half-life of α-amanitin.^3^

The mechanism of amatoxin-induced hepatotoxicity involves the entry of amatoxins into the hepatocyte through OATP1B1, an organic anion transporting polypeptide encoded by SLCO1B1, the expression of which is restricted to the basolateral membrane of hepatocytes.^4^^,^^5^ This has 2 direct consequences: (1) Because OAT1B1 is not expressed by kidney epithelial cells, other drug transporters are likely responsible for the entry of amatoxins including α-amanitin, into tubular cells. (2) Consequently, silibinin, which selectively inhibits OAT1B1, is ineffective in protecting tubular cells from the toxic effects of amatoxins, as evidenced with the development of ATI despite its administration. Notably, all therapies with the highest level of evidence for liver protection selectively target OAT1B1; in addition to silibinin, these include benzylpenicillin and silymarin. Therefore, it is critical to identify the proximal tubular transporters involved in amatoxin uptake by kidney tubular cells both at the basolateral (organic anion transporter, organic cation transporter) and apical membrane (multidrug and extrusion and toxin extrusion transporter). Doing so could pave the way for the use of existing drugs (eg, cimetidine, probenicid) or the development of novel tubular transport inhibitors.

The entry of the amatoxin into the hepatocyte is followed by inhibition of RNA polymerase II, resulting in an impediment of mRNA synthesis. The end result is cell death via p53-dependant apoptosis.^6^ Despite a high incidence of AKI ranging from 10% to 18% of *Amanita phalloides* poisoning cases, with partial or no recovery in 25% of these cases, the pattern of kidney damage has yet to be thoroughly investigated.^2^ Only a handful of case reports have highlighted ATI on biopsy, predominantly affecting the proximal tubule. In these cases, recovery was less frequent, and some patients progressed to end-stage kidney disease.^7^, ^8^, ^9^ Likewise, experimental studies have been confined to reports highlighting acute tubular necrosis, kidney inflammation and oxidative stress.^10^

Ultrastructural mitochondrial damage is a hallmark of various forms of toxic-induced ATI.^11^^,^^12^ Herein, histopathological investigations unraveled clear evidence of ultrastructural mitochondrial damage on optical microscopy, further corroborated by electronic microscopy.

**Figure 5:**
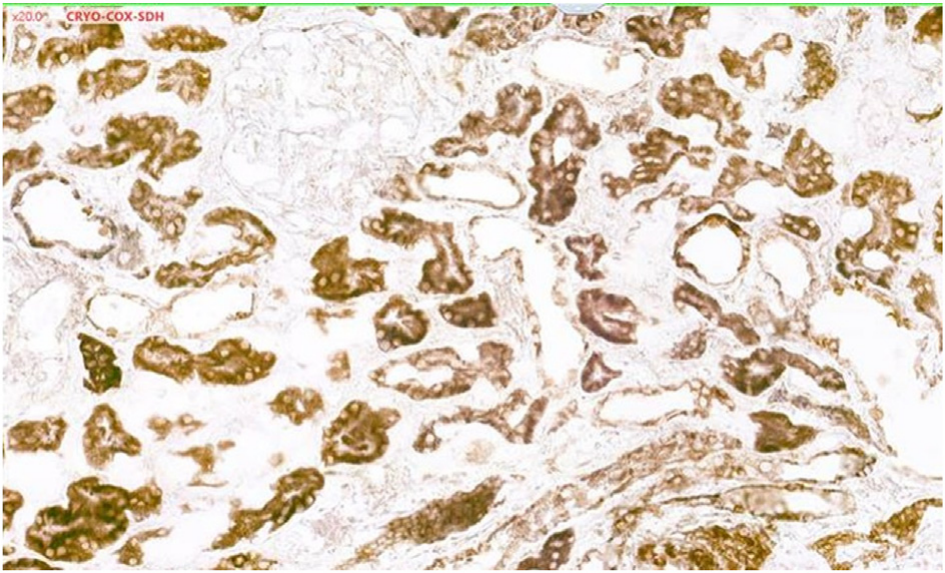
Decreased TOM20 kidney tubular expression after *Amanita phalloides* exposure. Comparison of TOM20 staining fluorescence intensity per proximal tubular cell in patient biopsies. ∗∗∗∗*P* < 0.0001.

Next, the expression of the key mitochondrial protein TOM20 was found to be diminished on histopathological protein-specific immunofluorescence (Figure 5). TOM20 is a major subunit of the TOM40 mitochondrial import complex which expression has been shown to be decreased in hepatocyte in in vitro models of α-amanitin toxicity.^6^^,^^13^ As mitochondrial damage is not only a hallmark of toxic ATI, but also a major pathway in ischemic acute tubular necrosis, we then compared TOM20 expression in our case with control patients displaying ischemic acute kidney of similar magnitude. Accordingly, the expression of TOM20 was significantly diminished in patients with Amanita poisoning, including after semiautomated quantification of TOM20-specific immunofluorescence.

Finally, drawing on tenofovir-induced kidney injury, representing another well-substantiated model of mitochondrial injury with uncertain renal recovery, we postulated whether patients with α-amanitin poisoning and AKI would yield a similar pattern of decreased succinate COX expression.^14^ In line with this hypothesis, electron microscopy showed a pervasive loss of cristae, the sites of oxidative phosphorylation. However, we did not find a significant decrease in cytochrome c oxidase activity, as assessed by histoenzymology, suggesting preserved mitochondrial IV complex function. Whether maintained COX activity reflects sublethal exposure to α-amanitin (the patient recovered a near normal estimated glomerular filtration rate) or indicates that a distinct pathway is in play remains to be proven. Alternatively, in various models of tubular mitochondrial injury, impaired respiratory function, including decreased COX activity, has been found to coexist with preserved TOM20 expression.^15^ Reciprocally, mitochondria derived from p53 knockout mice exhibited decreased TOM20 expression, whereas COX activity was unchanged.^16^ Taken together, these results suggest that our findings may not solely stem from experimental variability but may reflect independent pathophysiological pathways. In the face of diminished mitochondrial TOM20 import pore expression, we surmise that the translocation of p53 from the cytoplasm inside the mitochondria resides in its well-established interaction with Bcl2.

**Figure 6:**
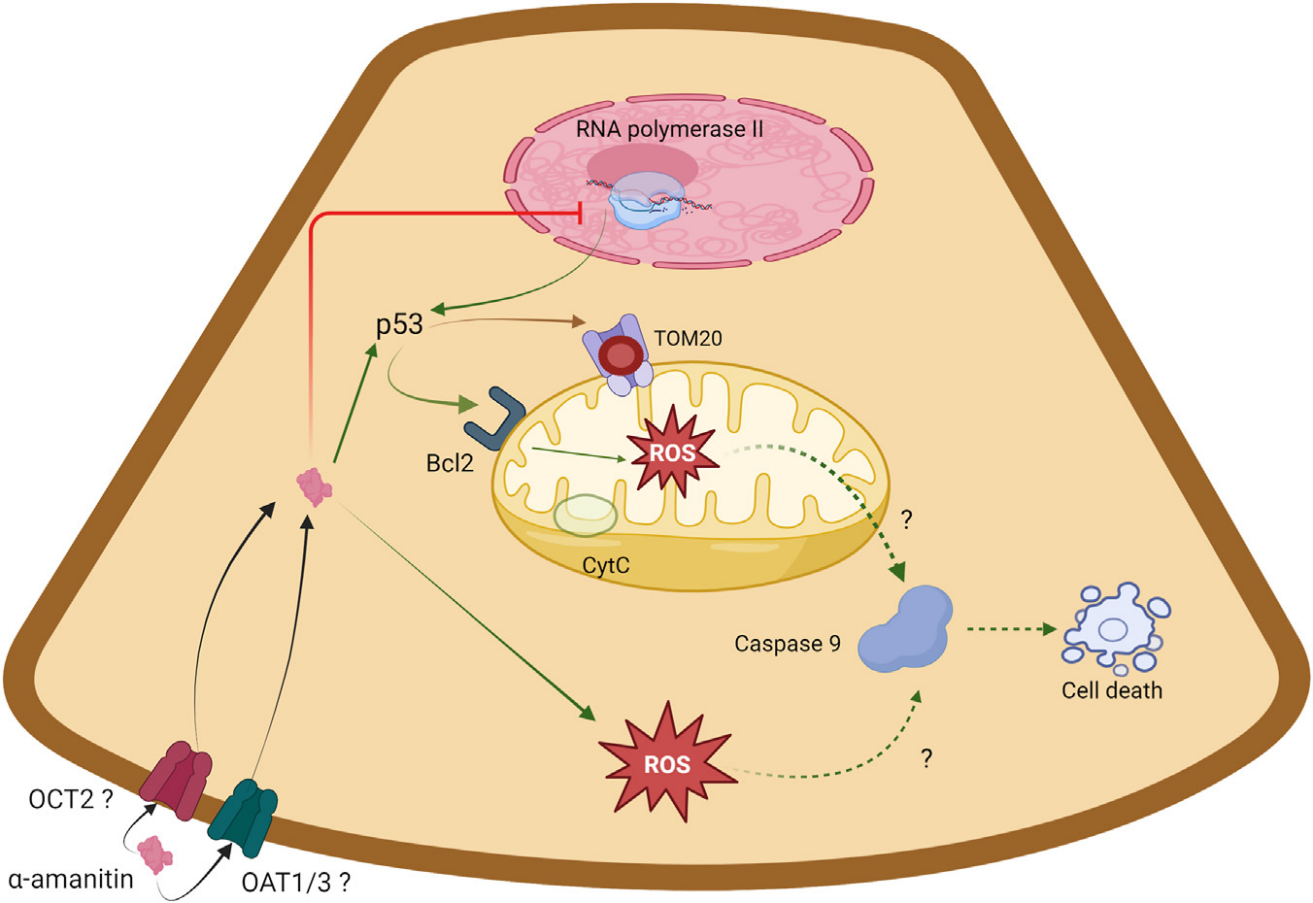
Hypothetical mechanisms of α-amanitin-induced proximal tubular cell toxicity. The exact entry route of α-amanitin into proximal tubular cells remains uncertain. However, based on in vitro hepatocyte models and our experimental results, we propose the following sequence of events: (1) Inhibition of RNA Polymerase II: α-Amanitin is known to inhibit RNA polymerase II, leading to the accumulation of p53 in the cytosol. (2) Mitochondrial Damage: Mitochondrial dysfunction is a central feature. TOM20 expression is reduced, although cytochrome c levels remain conserved. The intramitochondrial translocation of p53, believed to be mediated by Bcl-2, triggers the generation of reactive oxygen species (ROS) within mitochondria. (3) ROS Production in the Cytosol: α-Amanitin may also directly induce ROS production in the cytosol, potentially activating the caspase cascade and initiating apoptosis. Image created in BioRender (Rafat, C. (2024) BioRender.com/y83l255).

Significantly, the adverse effects of α-amanitin is likely to extend beyond the mitochondria. α-Amanitin triggers the generation of reactive oxygen species and oxidative stress, including in kidney tissue.^17^ From this standpoint, early administration of N- acetylcysteine may have mitigated the effects of α-amanitin-induced reactive oxygen species, generated through multiple pathways, thus dampening some of the functional and histological features of α-amanitin-induced ATI (Figure 6).

In summary, this case serves as a stark reminder that AKI, alongside liver failure, is an integral part of *Amanita phalloides* poisoning clinical spectrum. α-Amanitin-related ATI represents yet another model whereby mitochondrial dysfunction affecting the proximal tubule fosters the transition from AKI to chronic kidney disease, thus providing a basis for its incomplete kidney function recovery.^18^ Although the pathways of α-amanitin-induced tubular toxicity are yet to be fully elucidated, our findings provide insights that may guide the development of future kidney-protective therapies—a particularly crucial pursuit, given the inefficiency of silibinin in protecting kidney function.

## Supplementary Materials

Supplementary File (PDF) Item S1
