# Supplementary material for: Is Amanita phalloides Nephrotoxicity due to Mitochondrial Toxicity?
Source: Kidney Med. 2024 Dec 24;7(3):100952. doi: 10.1016/j.xkme.2024.100952 (PMC11835023; doi:10.1016/j.xkme.2024.100952)
Supplement: Supplementary File (PDF) — Item S1 [file mmc1.pdf]

Item S1: Appendix: **TOM 20 immunofluorescence and quantification**

Kidney biopsies were fixed in formalin for 4 hours and embedded in paraffin. Sections (3-µm thick) were processed for immunofluorescence. Biopsies' sections were deparaffinized and incubated for 20 min in the target retrieval solution pH6 (S2369, Dako, Agilent Technologies, Santa Clara, CA) at 100°C in a pressure cooker and blocked in PBS-0.1% Triton containing 10% bovine serum albumin. Sections were incubated overnight in a humidified chamber at 4°C with anti-Tom20 (1:100, D8T4N, Cell signaling), anti-megalin (1:2000, homemade) antibodies. The following day, after washings in PBS, sections were incubated for 1h at room temperature with Alexa Fluor 594-conjugated donkey anti-rabbit IgG secondary antibody and Alexa Fluor 488-conjugated chicken anti-goat IgG secondary antibody, each diluted at 1:500 in PBS 3% bovine serum albumin. Nuclei were then counterstained with DAPI (1:4000, 62,248, Thermo Fisher Scientific, Waltham, Massachusetts) for 5 min. Images were acquired using the slide scanner Axioscan Z1 (Carl Zeiss, Jena, Germany) at a 40x magnification. Image analysis was conducted using QuPath software (*Bankhead, P. et al. QuPath: Open source software for digital pathology image analysis. Scientific Reports (2017*). All cells were identified through DAPI staining of their nuclei using QuPath's Cell detection tool. Among the detected cells, only the proximal tubular cells were distinguished based on their positive megalin staining (green). Subsequently, the red fluorescence intensity corresponding to Tom 20 staining.
